# Person, Provider, Practice and Policy—P4: A framework for two-way learning and aboriginal community-led health promotion and evaluation

**DOI:** 10.1016/j.dialog.2026.100302

**Published:** 2026-04-10

**Authors:** Tracy McRae, Janella Isaac, Juli Coffin, Jonathan R. Carapetis, Roz Walker, Asha C. Bowen

**Affiliations:** a School of Medicine, University of Western Australia, Perth, WA, Australia; b Wesfarmers Centre of Vaccines and Infectious Diseases, The Kids Research Institute Australia, Nedlands, WA, Australia; c Ardyaloon Community, Kimberley, WA, Australia; d Ngangk Yira Institute For Change, Murdoch University, Perth, WA, Australia; e Department of Infectious Diseases, Perth Children's Hospital, Nedlands, WA, Australia; f Menzies School of Health Research, Charles Darwin University, Darwin, NT, Australia; g University of Notre Dame, Fremantle, WA, Australia

**Keywords:** Aboriginal health, Health promotion, Evaluation, Two-way learning partnerships

## Abstract

Research in Aboriginal communities starts where the community is at and works collaboratively and culturally responsively to the priorities and rhythms of the communities. The See Treat Prevent (SToP) Trial is the first clinical study to incorporate a holistic approach to reducing skin infections. This approach enabled the worldviews of Aboriginal people at the local community level to govern the empowerment approach underpinning the co-design of healthy skin resources and an evaluation framework. This manuscript reports the process of working on Country with Aboriginal communities to facilitate the restoration of Indigenous knowledge systems that have historically been excluded from health research, programs and policies.

**Methods:**

Yarning methodology was a approach for embedding Aboriginal worldviews within the Person, Provider, Practice and Policy (P4) evaluation framework showing the bi-directional interrelationships between community members, providers, and policies that affect health and wellbeing of Aboriginal people at the local community level. Importantly, the P4 model is a practical framework for considering the impact of policy and distance in real-world circumstances.

**Conclusion:**

Addressing the high prevalence of skin infections in Aboriginal children living in Kimberley communities requires empowerment approaches to include the voices of Aboriginal people living on Country. Doing so should not be a universal approach but a specific approach embedding local community culture and context to enable self-determination for Aboriginal communities. Health interventions that do not allow for transparency between community interrelationships will be difficult to implement or capture the important system inequities that often go unnoticed. It is anticipated that P4 can provide real opportunities for understanding health behaviours, not only at the Person level but also at the Provider, Practice and Policy levels to influence change.

## 1 Introduction

Research in Aboriginal communities starts where the community is at and works collaboratively and responsively to the priorities and rhythms of the communities [1]. Allowing sufficient time to build authentic relationships and partnerships with communities is crucial for health initiatives [2]. In doing so centralises Aboriginal epistemologies, ontologies and axiologies [3]—Aboriginal ways of knowing, being and doing [4] that consider the broader, social and emotional wellbeing, cultural and historical aspects impacting Aboriginal and Torres Strait Islander people's health. According to the National Aboriginal Community Controlled Health Organisation (NACCHO) [5]:

Aboriginal health means not just the physical well-being of an individual but refers to the social, emotional, and cultural well-being of the whole Community in which each individual is able to achieve their full potential as a human being thereby bringing about the total well-being of their community. It is a whole of life view and includes the cyclical concept of life-death-life.

(para 3)

Connection to Country is significant for the overall social and emotional wellbeing of Aboriginal and Torres Strait Islander people [5], [6], [7]. For Aboriginal people, ‘land was not owned; one belonged to the land’ ([7] p4). Land represented a deep spiritual connection. The Dreaming creation stories validated boundaries, and spiritual ancestors created and shaped local areas [6]. In the Kimberley region of Western Australia (WA), colonisation forced the displacement of Aboriginal people from their homelands and the removal of children from families [6]. The ongoing trauma experienced from legislation that enabled this displacement continues to contribute to the health inequities and high burden of disease, including skin infections experienced by Aboriginal people [6], [7]. Aboriginal children living in the Kimbeley are 15 times more likely to be hospitalised for a skin infection compared to non-Aboriginal children [8]. Globally, high rates of skin infections are reported from tropical, Pacific regions such as Fiji [9] and Samoa [10]. However, Australian Aboriginal children continue to experience the highest reported rate of skin infections globally for the past four decades [11]. Understanding the confounding factors contributing to the heavy burden of skin infections, such as access to functional plumbing, laundry facilities, and suitable housing for the often tropical, or harsh remote environments is fundamental for reducing the burden of skin infection through holistic approaches of treatment and prevention [12]. Furthermore, skin infections are often normalised [13] and go untreated which can lead to serious ongoing illnesses such as acute rheumatic fever and rheumatic heart disease [14]. Reducing the burden of skin disease is a public health priority [13].

### 1.1 .The See Treat Prevent (SToP) Trial—a hybrid skin health program

To address the high rates of skin infections in Aboriginal children living in the Kimberley region of WA, the See, Treat Prevent (SToP) Trial is the first skin clinical trial to incorporate a transdisciplinary approach with both biomedical components and prevention initiatives [15], [16], [17]. This hybrid approach challenges historical ‘treatment only’ clinical trials to encompass the broader social and environmental determinants specifically requested by Kimberley Aboriginal communities to address skin infections. Often under-represented and under-valued in biomedical settings [18], culture in health care celebrates the humility of holistic approaches for improving health and wellbeing and creates cultural security [19]. Medical anthropologist Napier and colleagues state:

The systematic neglect of culture in health is the single biggest barrier to advancement of the highest attainable standard of health worldwide (availability, accessibility, acceptability, and quality). The cultural practices of individuals and groups served should be better understood and acknowledged so that care systems can adjust practices in the interest of promoting wellbeing and reducing waste (p1630).

**Fig. 1:**
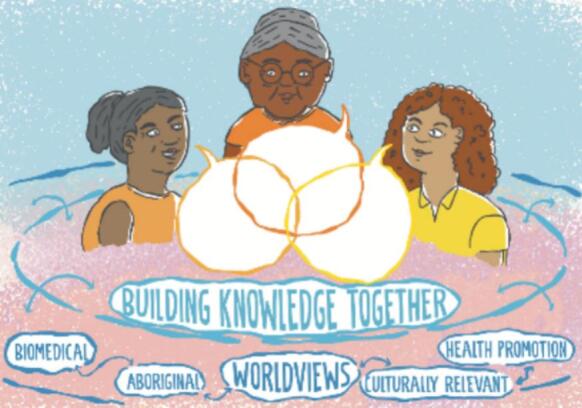
The intersection of Aboriginal and Western worldviews.

Culture and language in health care is now a high priority for public health [20], [21] and provides a space for narratives to complement numbers in addressing the disease burden. The intersection of the Prevention and biomedical components of the SToP Trial allowed the exploration of scientific and cultural knowledge in the quest to reduce skin infections. A cultural interface [22] is created where science and health promotion languages, and Aboriginal and non-Aboriginal worldviews intersect. The blending of these worldviews creates productive dialogues [23] to mitigate language discordance and make meaning of skin health directly with Aboriginal people, enabling an empowerment approach to health promotion (see Fig. 1).

### 1.2 Health promotion

The Ottawa Charter, developed at the inaugural international health promotion conference held in Canada [24], represents the principles and philosophy of health promotion as enabling and empowering all individuals to take control of their health. As a primary response to a new global public health movement at the time, the conference focused on the needs of countries undergoing significant social and political change, with similar consideration conveyed for all other regions. At its core, health promotion signifies advocacy, enablement, and mediation.

Globally, health promotion continues to evolve with new definitions for key concepts and new commitments focusing on community wellbeing, including ‘equitable distribution of resources, thriving and sustainability and societies that are resilient, build capacity and are prepared to overcome challenges’. [25].

### 1.3 Ethics in health promotion

In an Australian Aboriginal context, health interventions that have been developed ‘to help the well-being’ of Aboriginal people have invariably created more harm than good. To ensure history does not repeat itself, it is the moral and ethical responsibility of researchers and health practitioners to have Aboriginal and Torres Strait Islander people involved in all aspects of health research [26], [27] and health promotion [28], [29]. This commitment requires a shift in power dynamics to enable self-determination for Aboriginal and Torres Strait Islander people, as pointed out by Bond and colleagues [29]:

As both a moral and pragmatic endeavour, health promotion must include a more explicit discussion of power for its ethical basis. For this, a major reconfiguration of health promotion is required, involving a radical reworking of health promotion practitioner relationships with Indigenous people and communities. ‘We don't tell people what to do’ has particular significance for health promotion in this post-colonial setting, requiring the devolution and sharing of power with participating communities.

(p198)

Empowerment and behaviour change are synonymous with health promotion, and ‘health campaigns’ are used to influence individuals to change their behaviours and lifestyles to achieve better health outcomes [30]. In contrast to behaviour change campaigns ‘influencing’ people to change their health-related behaviours [31], [32], the ‘empowerment as a process’ approach provides individuals and communities as much control as possible over the change process [33]. These approaches to health promotion support McPhail and colleagues [28] perspective in that ‘ethical Indigenous health promotion necessitates Indigenous peoples’ control over the health promotion process and products, rather than the practitioners' or the service's control over Indigenous people or communities'(p198). In doing so, people have the freedom of choice to resume responsibility for their health and wellbeing. Tengland states that ‘given psychological conditions that include empathic listening, nonjudgmental attitudes, genuine participation on the part of professionals and enabling dialogical conditions, as well as external opportunities, the individuals or groups involved will empower themselves' (p143). Applying the Bardi ‘Oombarl Oombarl’ way of knowing, doing and being [4] in this work ensured Aboriginal people governed the health promotion process, centralising local community context, language, culture, and connection to Country healthy skin messages [34], [35].

Critics of empowerment as a process for health promotion consider this approach too time-consuming or ethically problematic when practitioners relinquish control over health promotion interventions [36]. These approaches will take time, but time is necessary for building trust and relationships with Aboriginal communities. Time also allows communities to *choose* the appropriate methods for developing, disseminating, and evaluating health messaging within the rhythm of community.

### 1.4 Unique worldviews of time and distance

Time has many meanings and definitions that vary across cultures. In Western cultures, time can be perceived as having a ‘linear’ form, based on the chronological notion that time is always moving in one direction (Geertz and Darnton 1973, as cited in Janca and Bullen [47]). In contrast, for Aboriginal people, time is multidimensional, it can resemble a pond – something you can glide through in every direction Furthermore, as quoted by Janca and Bullen [37], for Aboriginal people, time contains ‘no innate or inherent importance as such to an Aboriginal person; it is not adhered to and rarely directs an Aboriginal people but rather works for the person, family or community’ (p2).

In her seminal text, *Decolonising Methodologies: Research and Indigenous Peoples*, Linda Tuhiwai Smith critiques the concepts of time, space and distance constructed within a Western worldview, which have underpinned colonisation and informed policymaking. Smith highlights that the Industrial Revolution and evangelical movements reshaped Western perceptions of time, linking work with wealth and salvation. In contrast, Indigenous populations, uninfluenced by these historical events, applied time within cyclical rhythms. Smith noted, ‘Time is associated with social activity, and how other people organised their daily lives fascinated and horrified Western observers’(p273). The cyclical rhythm of daily life for Indigenous people made it difficult for Western observers to form a ‘complete idea of how the people divided their time’(p277). Western onlookers believed ‘native life was devoid of work habit’ (p274) and considered Indigenous people to be ‘lazy and indolent’. This discourse spanned colonisation and informed historical policymaking in Australia. The Western concepts of time are not benign, as they have a significant detrimental effect on Aboriginal lives.

In the Kimberley, there is a familiar discourse about time and distance. Everything runs on ‘Kimberley time’ and ‘everywhere is just up the road’ or ‘a little bit long way.’ While this discourse is often spoken in jest, there is a genuine need for flexibility when applying these concepts to research and health promotion.

This work valued Kimberley time and distance, numbers, and narratives to address skin health in real-world situations, spanning a four-year period ensuring trusting partnerships were developed and a deeper understanding of factors impacting skin health were better understood. This time facilitated space for co-designed health initiatives as well as opportunities to better understand how these initiatives could be better evaluated in a real-world context. Cribb [38] argues, ‘We need to be ready to analyse the ways in which models and theoretical frameworks get enacted and combined in practice, and especially the ways in which gaps easily open up between espoused models and real-world activities’(p203). Such considerations are particularly important in a cross-cultural context, as spending valuable time developing trust and relationships with communities can ‘come to nothing when measured against standard evaluations or funding limitations’. (p8) From a sociocultural perspective, the Aboriginal perception of time is valuable when developing mechanisms to assess [37] health promotion initiatives in Aboriginal communities.

This manuscript aims to contribute to the existing health promotion literature, describing the process of working in two-way partnerships with cultural mentors, Elders and community members On Country to centralise Aboriginal worldviews in the adaption of the Person, Provider, Practice and Policy (P4) evaluation framework applied in this work. The P4 framework extends on the work of Associate Professor Bednarczyk and colleagues [39], who developed the Practice, Provider, Patient (P3) framework. Bednarczyk et al. [39] applied P3 in a vaccination and colorectal cancer screening setting to recognise key activities across all three components essential for identifying barriers and enablers in prevention initiatives.

## 2 Methods

### 2.1 Study setting

This qualitative research project was situated within a broader PhD project in the ‘Prevent’ component of the SToP Trial conducted in nine remote Aboriginal communities spanning the East and West Kimberley region of WA. It values a NAACHO [5] collective approach to Aboriginal health referring to the social, emotional and cultural wellbeing of the whole community to ensure Aboriginal knowledges are grounded in ‘place’ and linked directly to the culture of community.

Applying a constructivist [40] approach to learn and embed Aboriginal knowledges within this work, I first acknowledge my worldview and positionality as a Kartiya (a common term in the Kimberley for a non-Aboriginal person) qualitative researcher. Guided by the Aboriginal (Bardi) ‘Oombarl Oombarl’ way of knowing, doing and being [4] as quoted below, this research is situated within an Indigenous Research Methodology (IRM) synonmous with the works of Aborignal scholars Karen Martin [4], Lester Rigney [41] and Linda Tuhiwai Smith [42], and also encompasses the concepts of Aboriginal Participatory Action Research (APAR) [1], [3]. This APAR approach facilitated a space for Aboriginal people to share realities and truths that have not always been well understood by non-Aboriginal researchers. In addition, this approach was fundamental to working within the rhythm of communities [1].

*So, I tend to work within that Oombarl Oombarl space, and I do tell people that I work with, ‘If you really want to find the truth, and what we're [Aboriginal people] trying to say, you have to take a step back and you've got to allow us to work within our own time’. [Aboriginal] people will share, people will engage, but if you're going to put pressure and expect people to work within a period—especially a short period. (Cultural Mentor, Ardyaloon Community).*

In seeking and learning the truths and realities for Kimberley Aboriginal communities, two-way learning [43] partnerships with Elders, community members and cultural mentors have guided this work. These two-way learning relationships revealed the history of Traditional Owner groups and the historical, political, and cultural determinants that continue to impact the lives of Aboriginal people living on homeland communities in the Kimberley [16], [17], [44], [45].

### 2.2 Data collection

Data was collected for this work using a combination of yarning methodology, semi-structed interviews, and personal journal notes. Developed by Kimberley Aboriginal scholar and social worker Professor Dawn Bessarab [46], yarning enabled reciprocal story sharing, authentic community leadership and a reflexive learning journey [47]. In the Australian Aboriginal context, yarning is a special way of storytelling that is central to Aboriginal ways of knowing, doing and being [4]. Semi-structured interviews were utilised when working with clinic and school staff, and stakeholders providing a diverse range of worldviews and experiences in the dataset.

### 2.3 Recruitment and participants

Participants for this study were recruited over a four-year period from 2019 to 2023 using both purposive and snowballing approaches [48]. More than 130 participants including community members, clinic and school staff, and stakeholders involved in the SToP trial were invited to contribute their knowledge, experiences and worldviews. All participants were aged 18 years or older. Recruiting was conducted face-to-face providing unique opportunities for the research team to go ‘On Country’ with Elders who shared personal stories and Dreamtime stories of the land. For Aboriginal people, Wright and colleagues (2023) explain ‘Country is a space of enunciation, one that cannot be detached from *Storying* because they are intertwined as a collective being.’ (1 p119) ‘On Country is not just a cultural experience, but historical and political – one of resistance. It is only after the On Country and the Shared Storying that it is possible to set the foundations for working together. Non-Aboriginal people must first have an understanding of relationships to Country and how our history was and continues to be written’.(1 p119).

All participants were informed of the research and offered a participant information sheet prior to providing their written informed consent and were assured of confidentiality and anonymity throughout the research project. When consent was provided, interviews were audio-recorded and saved as a digital recording in a deidentified format; for those yarning sessions or semi-structured interviews not recorded, handwritten notes were scribed.

### 2.4 Data analysis

All audio-recordings were transcribed verbatim and uploaded into QSR NVivo v12 [49] along with handwritten notes and relevant personal journal notes. Each transcript was assigned a code number to protect participant privacy, prior to an independent thematic analysis [48] being conducted. Iterative discussions regarding coding development, topic areas and emerging themes were conducted by the research team undertaking the analysis, until a consensus was reached. Themes and findings were corroborated and shared with communities and stakeholders.

### 2.5 Ethical approval

This project was approved by the health ethics review committees at the Child and Adolescent Health Service (Approval number RGS0000000584), the Western Australian Aboriginal Health Ethics Committee (Reference number: 819), University of Western Australia (Reference RA/4/20/4123), Catholic Education Western Australia (Reference number: RP2017/57) and Department of Education (Reference number: D18/0281633).

## 3 Results

**Fig. 2:**
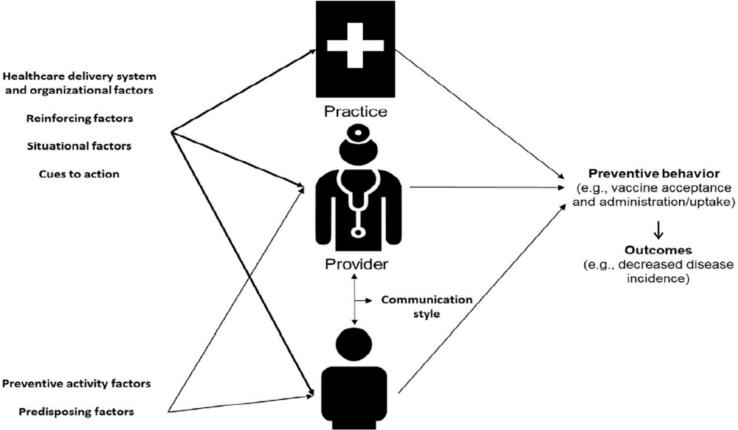
From Bednarczyk RA, Chamberlain A, Mathewson K, Salmon DA, Omer SB. Practice, provider- and patient-level interventions to improve preventive care: Development of the P3 Model. Prev Med Rep. 2018;11:131–38 (p134).

The three-component design of the P3 model (shown in Fig. 2) aligned with the transdisciplinary approach of the SToP Trial, where key activities were facilitated in school, clinic, and community settings, making it a practical tool for applying in a real-world context to understand skin infections and develop health promotion initiatives. The adapted P4 framework was co-designed and developed concurrently over a four-year period and include the worldviews of Aboriginal people living in Kimberley communities. Furthermore, the P4 framework highlights the critical bidirectional relationships and communications between community members, service provider organisations and policies that impact skin disease. Applying a critical lens and examining the three existing components in the P3 Model, the completed P4 framework is the final product of three iterations (see Fig. 5).

### 3.1 Practice, Provider, Patient (P3) behaviour change in prevention

As mentioned above, the framework applied in this work extends on the work of Associate Professor Bednarczyk and colleagues [39], who developed the Practice, Provider, Patient (P3) framework (see Fig. 2). In developing P3, Bednarczyk and colleagues used existing theories synonymous with behaviour change, health promotion and preventative care: the health belief model, the theory of planned behaviour, the social cognitive theory, the social-ecological model, and the systems of models of clinical preventative care. Underpinned by psychology, these theories explore human behaviours at the individual level and, to a lesser extent, the socio-ecological for evaluating behaviour change and prevention initiatives.

These theories are not explained in detail here; however, the conceptuality and practicality of the P3 model in real-world preventative care are significant. Encompassing key components from both the individual and broader ecological theories, the P3 model considers the individual, the socio-ecological, as well as the systematic factors underlying preventative care; for example, provider–patient communication, health care and organisational factors and preventive and predisposing factors influencing health behaviours. The P3 model has been used in Australia to design a multi-component intervention to promote antenatal care. With permission from Associate Professor Robert Bednarczyk, the P3 model was the foundation for developing the P4 framework.

#### 3.1.1 Stage 1: the first iteration

**Fig. 3:**
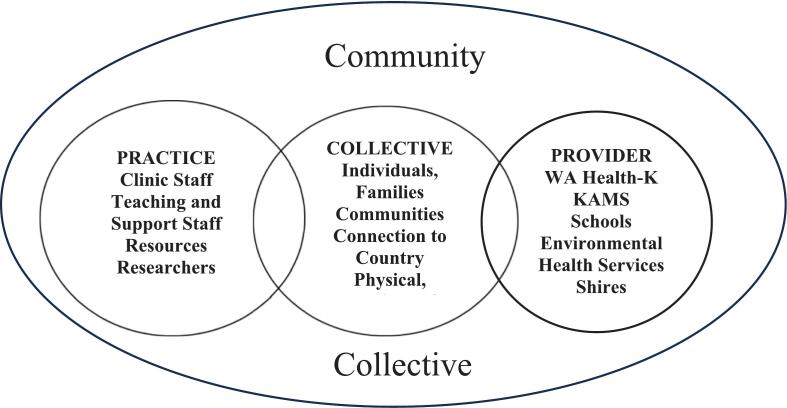
P3 adapted with permission from Bednarczyk RA, Chamberlain A, Mathewson K, Salmon DA, Omer SB. Practice, provider- and patient-level interventions to improve preventive care: Development of the P3 Model. Prev Med Rep. 2018;11:131–38. (p134).

The components in the P4 model were rearranged to reflect Aboriginal community context and interrelationships accurately. In the first iteration of P3, shown in Fig. 3, the Patient component was renamed ‘Collective’ to represent the NAACHO [5] holistic concept of health for Aboriginal people. Community service provider organisations are represented as the ‘Provider’ component, and the staff/practitioners are embedded within those organisations at the ‘practice’ level. Theoretically, this approach resonated with Aboriginal Kimberley community contexts; however, it did not show the bidirectional interrelationships between the three components or account for policymaking.

#### 3.1.2 Stage 2: the second iteration

**Fig. 4:**
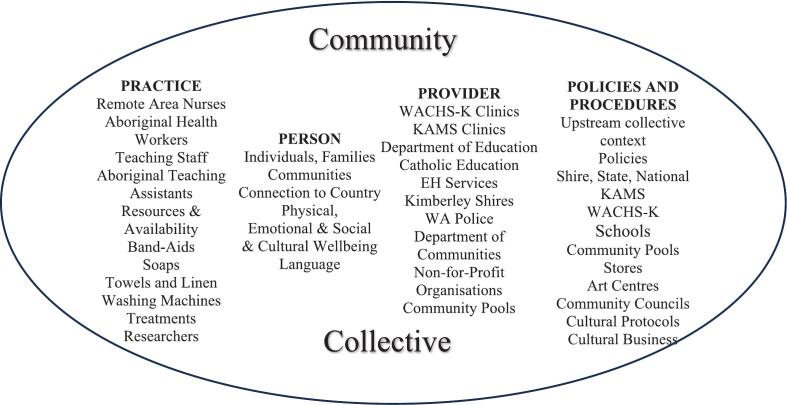
Person, Provider, Practice and Policy (P4) framework second iteration.

As this work progressed during the development of a community-led hip-hop video [35], and co-designed healthy skin books [34] the historical, social, and political factors impacting the cultural wellbeing of Aboriginal people living in homeland communities became more apparent. Resulting in the extension of the P3 model to include a ‘Policies and Procedures’ component (shown in Fig. 4) to reflect the impact of decision-making in communities contributing to health, particularly skin health for Aboriginal people living in Kimberley homeland communities as reported by McRae and colleagues [44].

**Fig. 5:**
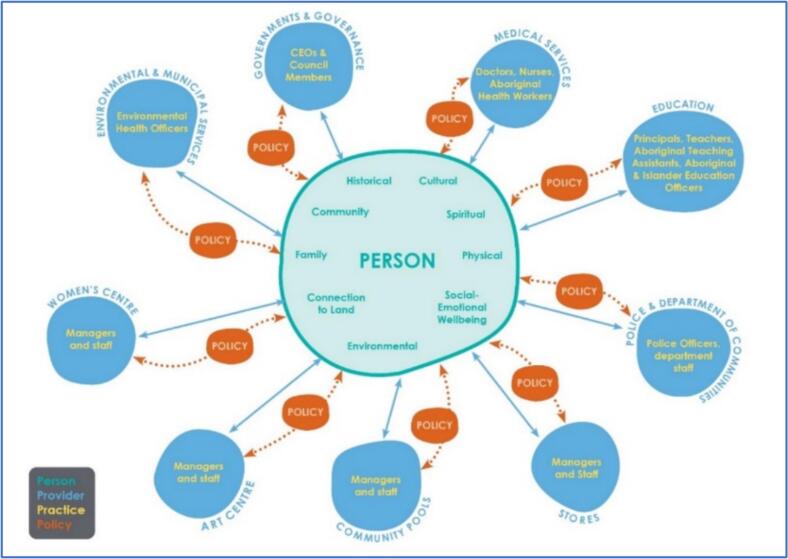
Person, Provider, Practice and Policy (P4) framework showing bidirectional relationships between all four components.

#### 3.1.3 Stage 3: the final iteration

Theoretically, the second iteration also worked in the real-world context to identify preventative and predisposing factors of health and wellbeing in homeland Aboriginal communities. However, the interrelationships between all four components were not obvious or transparent. For example, the policy component was visible but did not link back to communities; therefore, it is difficult to illustrate how policy affects Aboriginal people without showing the bidirectional interrelationships.

Reflecting on preventative and predisposing factors encompassing collective Aboriginal health shared by Elders and community members during yarning sessions and semi-structured interviews [16], [17], [44] Patient was recreated to ‘Person’ (in keeping with the ‘Ps’) and centralised Person within a collective sphere. This sphere captures the NAACHO [5] holistic health meaning, extending beyond skin health to encompass Aboriginal wellbeing. Yarning with Elders, community members, clinic staff and school staff also clarified which service provider organisations typically operated in Aboriginal communities as reported by McRae and colleagues [44]. These organisations are reflected in the Provider outer spheres with two-way arrows signifying the bidirectional interrelationships with Person. The Practice component has been situated within the Provider spheres to capture the staff and practitioners' roles within these service provider organisations. While local community members occupy some roles, the high turnover of fly-in-fly-out rostered staff in these organisations affects the relationships and rhythm of life in homeland communities [17], [44].

### 3.2 The inclusion of policy

Policy is critical for influencing health care and resources; however, existing individual and socio-ecological models do not capture the bidirectional interrelationship between person and policy to understand the impact of policy on health behaviours. The extension of P3 explores the relevance and appropriateness of adopting and recreating this theoretical framework to a P4 model to include or acknowledge policy within an Aboriginal community context. This work was important given that Western health programs, policies and evaluation frameworks in an Aboriginal context have not included Aboriginal people's voices, despite attempting to improve health outcomes.

Including policy in P4, provides new knowledge and processes to address existing structural inequities and improve collaborations between service provider organisations in Aboriginal homeland communities. Including policy enables the research process, particularly health promotion and behaviours, to be assessed across multiple levels encompassing policy at the proximal level of the people being affected. Policy invariably is designed and intended to benefit a particular population however historically policy has been ‘done to people’ not ‘with people.’ This is particularly true for Aboriginal people living in Kimberley communities to highlight the importance of two-way policy. Adding this dimension to the P4 model enables this work to be considered within the existing policy frameworks such as the National Partnership Agreement Closing The Gap [20] priority reforms and the WA Aboriginal Health and Wellbeing Framework [50]. These policies signify equity in policymaking to include Aboriginal voices in the decision-making. The WA Health Promotion Strategy [51] references the importance of community engagement and voices; however, it does not provide a practical application to assess the effectiveness and cultural responsiveness of health programs. There is now a crucial requirement and important opportunity to apply the P4 model in future health programs. Importantly, P4 incorporates Kimberley community voices as well as the concepts of time and distance significant for Aboriginal people which can be transferred more broadly.

## 4 Conclusion

Addressing the high prevalence of skin infections in Aboriginal children living in Kimberley communities requires empowerment approaches to include the voices of Aboriginal people living on Country. Doing so should not be a universal approach but a specific approach embedding local community culture and context to enable self-determination for each individual Aboriginal community [1]. Time, distance and flexibility are key aspects of Aboriginal community-led health promotion; operating to the rhythm of these communities is essential. Reconceptualising methodologies for health programs require deconstructing Western paradigms that value objectivity and impersonal approaches and replacing them with methods that enforce building trust and relationships. This endeavour will allow for a deeper understanding of what health and wellbeing mean for Aboriginal people so they can guide the process for their health outcomes.

The SToP Trial is the first healthy skin program to reduce skin infections through skin surveillance at school, treatment at health clinics and community-driven health promotion activities [15]. Underpinned by APAR [1], [3], Aboriginal stakeholders and researchers guided the SToP healthy skin project to centralise Aboriginal ways of knowing, doing and being [4]. The Bardi ‘Oombarl Oombarl’ way of knowing, doing and being [4] shaped this methodology. Being On Country and yarning with Elders and community members, coupled with two-way learning partnerships, enabled this work to be conducted in a culturally appropriate manner and to the rhythm of communities. In addition, being invited On Country was a rich learning experience to learn the truths and realities of Elders and community members that guided the process of developing community-led SToP Trial health promotion initiatives [44], including a hip-hop video and healthy skin books centralising Aboriginal worldviews. The process of developing community-led healthy skin resources has been reported elsewhere [34], [35], and are available via this link https://www.thekids.org.au/healthy-skin/resource-hub.

Taking the ‘Oombarl Oombarl’ approach to revise the P3 model into P4 required flexibility and time, which are critical to align with the rhythm of life in Kimberley Aboriginal communities. Importantly, the P4 model is a practical framework for considering the impact of policy and distance in real-world circumstances. Health interventions that do not allow for transparency between community interrelationships will be difficult to implement or capture the important system inequities that often go unnoticed. It is anticipated that P4 can provide real opportunities for understanding health behaviours, not only at the Person level but also at the Provider, Practice and Policy levels influencing change.

## CRediT authorship contribution statement

**Tracy McRae:** Writing – original draft, Methodology, Formal analysis, Data curation, Conceptualization. **Janella Isaac:** Writing – review & editing, Methodology. **Juli Coffin:** Writing – review & editing, Supervision. **Jonathan R. Carapetis:** Writing – review & editing, Supervision. **Roz Walker:** Writing – review & editing, Supervision, Methodology. **Asha C. Bowen:** Writing – review & editing, Supervision, Methodology.

## **Ethics**

This project was approved by the health ethics review committees at the Child and Adolescent Health Service (Approval number RGS0000000584), the Western Australian Aboriginal Health Ethics Committee (Reference number: 819), University of Western Australia (Reference RA/4/20/4123), Catholic Education Western Australia (Reference number: RP2017/57) and Department of Education (Reference number: D18/0281633).

## Funding

Funding was received from the 10.13039/501100000925National Health and Medical Research Council [NHMRC] (GNT1128950), Health Outcomes in the Tropical NORTH [HOT NORTH 113932] Indigenous Capacity Building Grant), and WA Health Department and Healthway grants contributed to this research. ACB receives a NHMRC Investigator Award (GNT1175509). TM receives a PhD scholarship from the Australian Centre for Elimination of Neglected Tropical Diseases (ACE-NTD), an NHMRC Centre of Excellence (APP1153727).

## Declaration of competing interest

The authors declare the following financial interests/personal relationships which may be considered as potential competing interests:

Tracy McRae reports financial support was provided by Australian Centre for Control and Elimination of Neglected Tropical Diseases. Asha C Bowen reports financial support was provided by 10.13039/501100000925National Health and Medical Research Council. If there are other authors, they declare that they have no known competing financial interests or personal relationships that could have appeared to influence the work reported in this paper.
